# Impact of Amyloid-β on Platelet Mitochondrial Function and Platelet–Mediated Amyloid Aggregation in Alzheimer’s Disease

**DOI:** 10.3390/ijms22179633

**Published:** 2021-09-06

**Authors:** Lili Donner, Tobias Feige, Carolin Freiburg, Laura Mara Toska, Andreas S. Reichert, Madhumita Chatterjee, Margitta Elvers

**Affiliations:** 1 Department of Vascular and Endovascular Surgery, Experimental Vascular Medicine, Medical Faculty and University Hospital Düsseldorf, Heinrich-Heine University Düsseldorf, 40225 Düsseldorf, Germany; Tobias.Feige@med.uni-duesseldorf.de (T.F.); Carolin.Freiburg@uni-duesseldorf.de (C.F.); LauraMara.Toska@med.uni-duesseldorf.de (L.M.T.); 2 Institute of Biochemistry and Molecular Biology I, Medical Faculty and University Hospital Düsseldorf, Heinrich-Heine University Düsseldorf, 40225 Düsseldorf, Germany; reichert@hhu.de; 3 Department of Cardiology and Angiology, Universitätsklinikum Tübingen, Medizinische Klinik III, 72076 Tübingen, Germany; madhumita.chatterjee@med.uni-tuebingen.de

**Keywords:** Alzheimer’s disease, platelets, mitochondria dysfunction, Aβ aggregation, cerebral amyloid angiopathy, ROS, GPVI, integrin

## Abstract

Background: Alzheimer’s disease (AD) is characterized by an accumulation of amyloid β (Aβ) peptides in the brain and mitochondrial dysfunction. Platelet activation is enhanced in AD and platelets contribute to AD pathology by their ability to facilitate soluble Aβ to form Aβ aggregates. Thus, anti-platelet therapy reduces the formation of cerebral amyloid angiopathy in AD transgenic mice. Platelet mitochondrial dysfunction plays a regulatory role in thrombotic response, but its significance in AD is unknown and explored herein. Methods: The effects of Aβ-mediated mitochondrial dysfunction in platelets were investigated in vitro. Results: Aβ40 stimulation of human platelets led to elevated reactive oxygen species (ROS) and superoxide production, while reduced mitochondrial membrane potential and oxygen consumption rate. Enhanced mitochondrial dysfunction triggered platelet-mediated Aβ40 aggregate formation through GPVI-mediated ROS production, leading to enhanced integrin αII_b_β_3_ activation during synergistic stimulation from ADP and Aβ40. Aβ40 aggregate formation of human and murine (APP23) platelets were comparable to controls and could be reduced by the antioxidant vitamin C. Conclusions: Mitochondrial dysfunction contributes to platelet-mediated Aβ aggregate formation and might be a promising target to limit platelet activation exaggerated pathological manifestations in AD.

## 1. Introduction

The most prevalent form of dementia is Alzheimer’s disease (AD). AD is characterized by the pathological hallmarks of abnormal accumulation of amyloid β (Aβ) peptides in the brain [1]. One of the earliest pathological alterations in AD is the dysfunction of mitochondria [2]. Mitochondrial abnormalities, such as impaired mitochondrial dynamics (increased fission and reduced fusion), altered morphology and mitochondrial gene expression, increased free radical production and lipid peroxidation, reduced cytochrome c oxidase (COX) activity and ATP production, are typical characteristics of AD. Mitochondrial dysfunction results from these morphological and metabolic alterations during the progression of AD [2,3,4,5]. Soluble Aβ enters mitochondria and is responsible for mitochondrial dysfunction that contributes to phosphorylation of tau, increased formation of free radicals, mtDNA damage and interaction of Aβ with Drp1, Aβ-binding alcohol dehydrogenase (ABAD) and cyclophilin D (CypD), loss of cytochrome c oxidase (COX) activity, impaired gating of the mitochondrial permeability transition pore and loss of membrane potential. In the presence of Aβ, mitochondria are reduced in size due to excessive mitochondrial fragmentation and reduced mitochondrial fusion, but increased in numbers [6,7,8,9,10]. Increased levels of Aβ in the cytoplasm of AD neurons also lead to reduced levels of parkin and PTEN-induced putative kinase1 (PINK1) leading to the inability to clear damaged mitochondria (mitophagy) and other cellular debris from neurons [11]. Persisting mitochondrial dysfunction contributes to synaptic dysfunction because declined mitochondrial biogenesis leads to reduced ATP levels that is essential for delivery of neurotransmitters by synaptic vesicles to the synapse [12]. Furthermore, the loss of COX activates apoptotic pathways, leading to the loss of neurons in the central nervous system [6]. As a compensatory mechanism for dysfunctional energy metabolism, mitochondrial-encoded genes are upregulated in AD transgenic mice [10]. While mitochondrial dysfunction plays a central role in the pathogenesis of AD, the dysregulated mitophagy is causative in worsening disease pathology/severity in AD.

Platelets expose amyloid precursor protein (APP) at the platelet membrane and include all the necessary enzymes for the generation of different Aβ peptides from APP in their alpha granules [13]. Therefore, platelets are a source of Aβ peptides in blood [14]. Furthermore, apoptotic platelets are able to incorporate oligomeric Aβ40 [15]. Different studies have identified pathological alterations in isolated platelets from AD patients, such as a decreased amyloid protein precursor ratio and an increased activity of β-secretase leading to Aβ production [16,17]. Moreover, AD patients exhibit increased basal activation of platelets, denoted by enhanced surface expression of P-selectin and presence of activated integrin α_IIb_β_3_ [18]. Pre-activated platelets in the circulation are also observed in the AD transgenic mouse model APP23. These platelets adhere to amyloid deposits in cerebral vessels causing vessel occlusion [19]. A subpopulation of coated platelets with high procoagulant activity is elevated in AD patients and correlates with the progression of AD [20]. Interestingly, apart from their ability to generate Aβ peptides, predominantly Aβ40, platelets also modify soluble synthetic Aβ40 into toxic Aβ aggregates in vitro [15]. Platelet-specific receptors, namely integrin α_IIb_β_3_ and collagen receptor glycoprotein (GP)VI, were identified as direct binding partners of Aβ40 at the platelet membrane that contribute to platelet activation and aggregation of Aβ40 peptides [21,22,23].

Although platelets contain only five to eight mitochondria per cell [24], they play an important role in energy metabolism and ATP production in platelets. Moreover, mitochondria are also involved in platelet activation and apoptosis [25]. Previous studies have provided evidence for altered mitochondrial function in platelets from AD patients. Compared to platelet mitochondria from healthy volunteers, platelet mitochondria from AD patients exhibit decreased maximal capacity of the electron transport system and reduced respiration rates [26,27]. In addition, platelets from AD patients show decreased COX activity, which is associated with ROS overproduction [28].

Despite the extensive research work in the last decades and the progress that has been made to understand the pathophysiology of AD, there are many open questions in understanding the molecular basis of the disease. However, it is well accepted that AD is a multi-factorial neurodegenerative disease and there is still no drug or therapy available to delay or even prevent dementia in patients with AD. In recent years it has been increasingly recognized that platelets can not only serve as a biomarker for the disease but also substantially contribute to the progression of AD. However, the impact of platelets in AD progression is not fully understood. Therefore, the present study aimed to investigate the effect of Aβ40 peptides on platelet mitochondrial dynamics and its consequences for platelet activation and Aβ40 aggregate formation.

## 2. Results

### 2.1. Effects of Aβ40 on Mitochondria in Platelets

**Figure 1:**
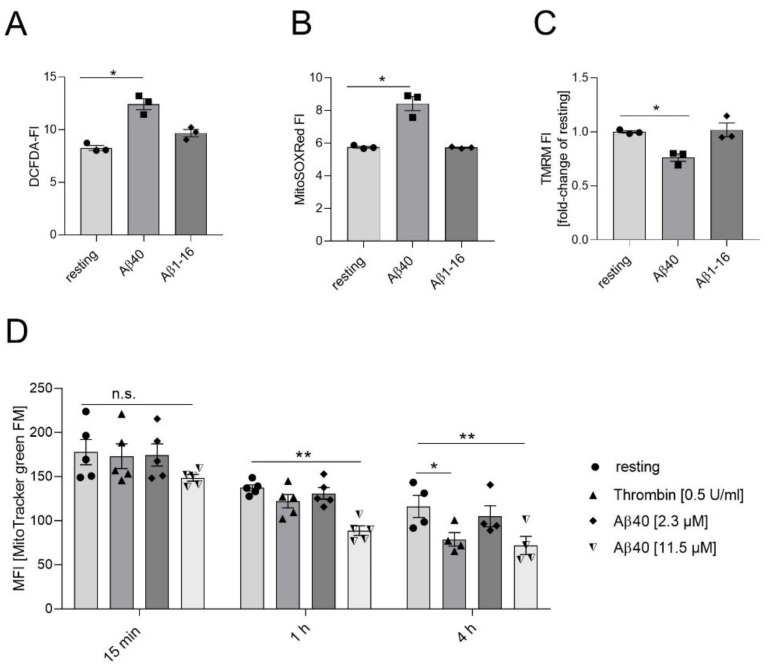
Impact of Aβ on mitochondrial functions in platelets. (**A**) Washed human platelets were incubated for 24 h with 11.5 µM Aβ40 and 11.5 µM Aβ1-16 (as control). Generation of ROS was reported as mean fluorescence intensity of DCF (n = 3). (**B**) MitoSOXRed™-loaded platelets were incubated with 5 µM Aβ40 and 5 µM Aβ1-16 for 30 min and the generation of superoxide was measured as mean fluorescence intensity (n = 3). (**C**) Depolarization of the platelet mitochondrial membrane upon 5 µM Aβ40 and 5 µM Aβ1-16 was observed by decreased TMRM fluorescence intensity (n = 3). (**D**) The mitochondrial dye, MitoTrackerTM green FM, was added to platelets to determine the release of mitochondria upon Aβ40 stimulation (n = 4–5). Compared to control (resting). (**A**–**D**) All samples were measured by flow cytometry. Bar graphs depict mean values ± SEM. All analyses were performed using one-way ANOVA and Dunnett’s multiple comparisons post-hoc test. ** *p*< 0.01; * *p*< 0.05. n.s.: not significant.

Generation of reactive oxygen species (ROS) plays an influential role in pathophysiological platelet functions [29]. To investigate the effect of Aβ40 on intracellular ROS production in platelets, we measured ROS by flow cytometry using the dye DCF-DA. The cellular ROS level in platelets from healthy donors was significantly increased upon stimulation with Aβ40 (Figure 1A). In contrast, the stimulation with Aβ1-16 (Aβ16, used as negative control) did not lead to increased ROS levels. To determine the impact of Aβ40 on mitochondrial superoxide production, we used the mitochondrial localized ROS sensitive dye MitoSOX-Red. The generation of mitochondrial superoxide was increased upon Aβ40 stimulation and unaltered by Aβ16 (Figure 1B). Using the cationic dye TMRM, we determined whether or not stimulation of platelets with Aβ40 led to a loss of the mitochondrial transmembrane potential (Δψm) (Figure 1C). As shown in Figure 1C, the incubation of platelets with Aβ40 led to significantly reduced Δψm in platelets, whereas Aβ16 showed no effect.

Bourdeau and colleges have shown that activation of platelets induces release of mitochondria to promote inflammatory response [30]. Using a mitochondria-selective, yet membrane potential insensitive, fluorescent dye (MitoTracker™ green FM, Invitrogen), we detected that thrombin activation of platelets led to a reduced mean fluorescence intensity (MFI) consistent with the release of mitochondria after 4 h of incubation (Figure 1D). By contrast, platelets started to release mitochondria upon stimulation with 11.5 µM Aβ40 already after 1 h and continued till 4 h of incubation, while no effects could be seen with lower concentrations of Aβ40 (2.3 µM) (Figure 1D).

### 2.2. Reduced Mitochondrial Respiration in Platelets Following Aβ40 Treatment

**Figure 2:**
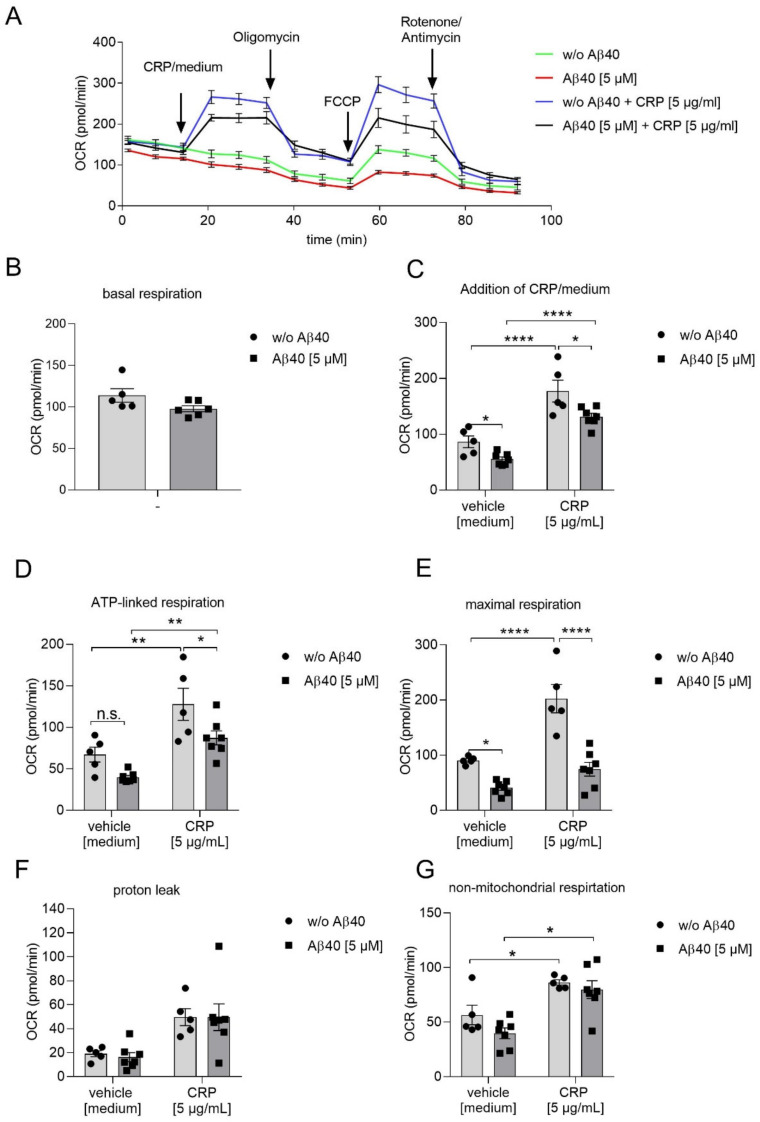
Determination of oxygen consumption rate (OCR) in human platelets upon treatment with Aβ40. (**A**) Determination of the oxygen consumption rate (OCR) after injection of indicated chemicals at indicated time points using a Seahorse XF24 analyzer. (**B**) Basal respiration, (**C**) respiration after addition of collagen-related peptide (CRP) or vehicle (medium), (**D**) ATP-linked respiration, (**E**) maximal respiration, (**F**) proton leak and (**G**) non-mitochondrial respiration. (**A**–**G**) Data represent mean ± SEM from n = 5–7 donors, two-way ANOVA with Holm-Sidak’s multiple comparisons test, **** = *p* < 0.0001; ** = *p* < 0.01; * = *p* < 0.05. w/o: Without; n.s.: Not significant.

Using the Seahorse Extracellular Flux Analyzer, platelet mitochondrial respiration was investigated after incubation with soluble Aβ40 by measuring the oxygen consumption rate (OCR). Aβ40 or vehicle (medium) was added to platelets for 30 min before measurement. The basal OCR in platelets in the presence of Aβ40 was comparable to resting platelets (control) after 30 min (Figure 2A,B). After injection of collagen related peptide (CRP) to activate the major collagen receptor GPVI, we found a general stimulation of respiration rates (Figure 2A,C). Importantly, a significant relative reduction of OCR in platelets was evident always when Aβ40 was added irrespective of the addition of CRP. A significant reduction of the OCR over time was also found when platelets were incubated with Aβ40 compared to unstimulated platelets (Figure 2A,C). To measure ATP-linked respiration, the ATP synthase inhibitor oligomycin was added subsequently. Again, CRP induced an enhanced OCR compared to non-stimulated platelets. However, in the presence of Aβ40, CRP-induced OCR was significantly reduced (Figure 2A,D). Afterwards, the proton ionophore and uncoupler FCCP was injected to measure maximal respiration (Figure 2A,E). As expected, the injection of FCCP led to increased respiration in CRP-stimulated platelets. Again, the presence of Aβ40 reduced maximal respiration in both resting and in CRP-stimulated platelets. Moreover, the increase of CRP-induced maximal respiration was reduced to basal (resting) levels when platelets were incubated with Aβ40 (Figure 2A,E). Proton leak across the inner mitochondrial membrane was not changed in the presence of Aβ40 (Figure 2A,F). Non-mitochondrial respiration ascertained using the mitochondrial complex inhibitors Antimycin A and Rotenone was not altered between groups (Figure 2A,G). Taken together, these results demonstrate that Aβ40 negatively impacts mitochondrial respiration in resting and CRP-stimulated platelets.

### 2.3. Impact of Extracellular Aβ40 on Mitochondrial Proteins

**Figure 3:**
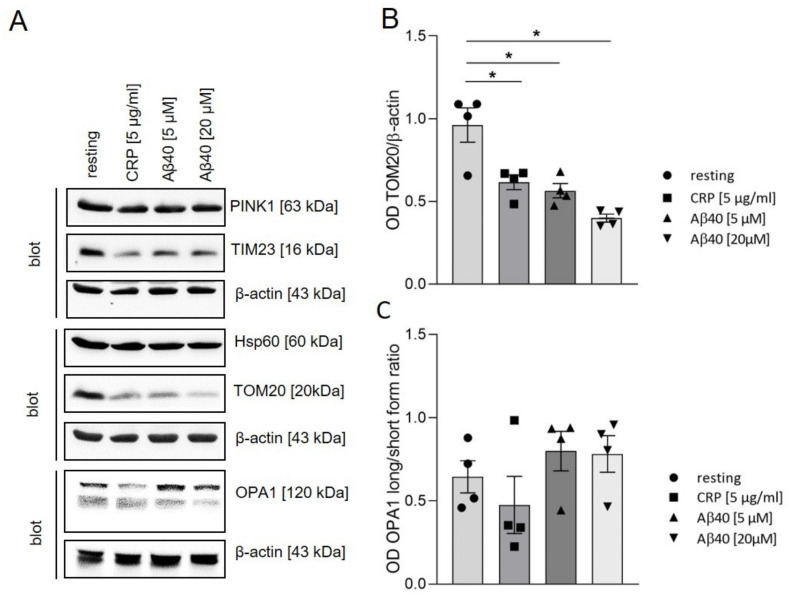
Expression levels of mitochondrial proteins in platelets upon Aβ40 stimulation. (**A**) Human platelets were stimulated with 5 or 20 µM Aβ40 or 5 µg/mL CRP for 2 h. Using Western blot analysis, the expression levels of mitochondrial proteins were detected as indicated. β-actin served as loading control. (**B**,**C**) The intensity of bands was analyzed with ImageJ software. Data represent mean value ± SEM (n = 4). All analyses were performed using one-way ANOVA and Dunnett’s multiple comparisons post-hoc test. * = *p* < 0.05.

To investigate the impact of extracellular Aβ40 on mitochondrial proteins, such as PTEN-induced putative kinase 1 (PINK1), translocase of the inner membrane 23 (TIM23), 60 kDa heat shock protein (Hsp60), optic atrophy-1 (OPA1) and translocase of the outer membrane 20 (TOM20) in platelets, we incubated platelets with different concentrations of Aβ40 for 2 h and analyzed protein expression by Western blot. Non-stimulated platelets were used as negative and CRP-stimulated platelets were used as positive control. The incubation with different concentrations of Aβ40 for 1 h did not induce alterations in the level of the examined proteins (Figure S1). After incubation of platelets with Aβ40 for 2 h, the protein levels of PINK1, TIM23 and Hsp60 were comparable to that of resting platelets (Figure 3A and Figure S2). However, the protein level of TOM20 was significantly reduced when human platelets were stimulated with intermediate and high concentrations of Aβ40 as well as with CRP (Figure 3A,B), suggesting that downregulation of TOM20 is not due to Aβ40 toxicity but due to platelet stimulation. Determination of the ratio of long OPA1/short OPA1 revealed no significant alterations and a trend towards a reduced ratio was only observed when platelets were stimulated with CRP. Addition of Aβ40 did not significantly alter the ratio of long OPA1/short OPA1.

### 2.4. Inhibition of Complex III Leads to Enhanced Platelet Mediated Aβ Aggregate Formation In Vitro

**Figure 4:**
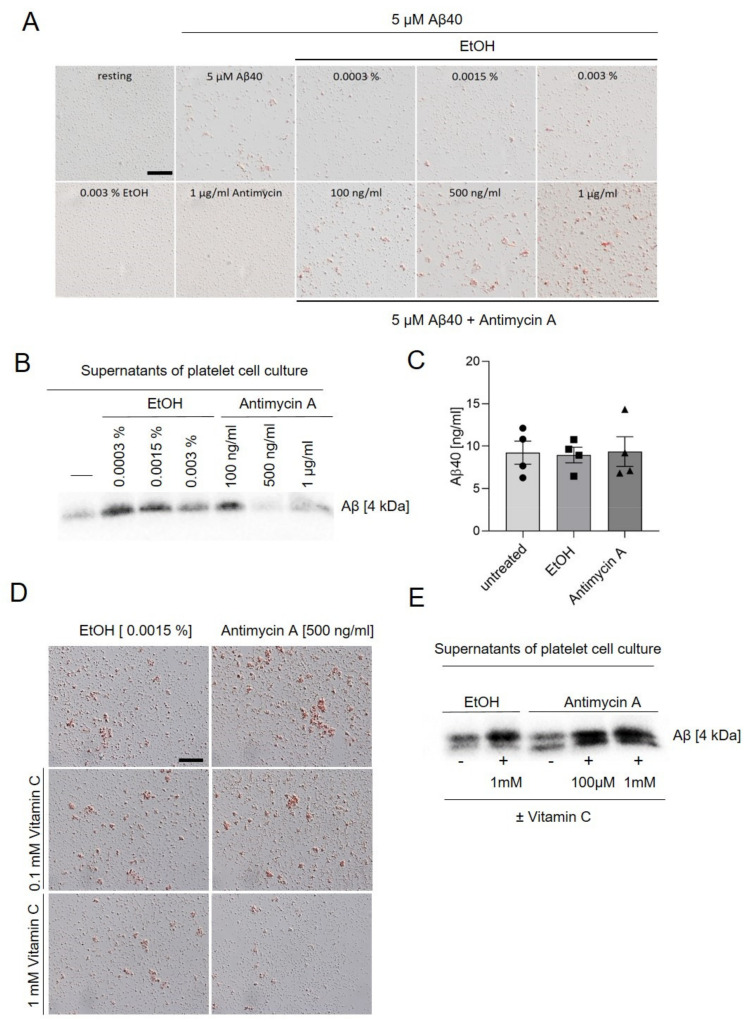
Effects of antimycin A and the antioxidant vitamin C on platelet-mediated Aβ aggregate formation. (**A**) Representative images of congo red-stained Aβ deposits in platelet culture. Platelets were incubated with 5 µM of soluble synthetic Aβ40 and different concentrations of antimycin A for three days at 37 °C and 5% CO_2_. EtOH served as the control (vehicle). Scale bar, 50 µm. (**B**) Quantification of remaining soluble Aβ40 in the supernatant of platelet culture using Western blot. (**C**) Isolated platelets were incubated in the absence or presence of antimycin A (500 ng/mL) for 24 h at 37 °C. EtOH (0.0015%) served as solvent control for antimycin A. Aβ40 levels were determined using ELISA (n = 4). (**D**) Platelet culture after incubation with soluble, synthetic Aβ40 for three days. Where indicated, platelets were incubated with antimycin A (500 ng/mL) or antimycin A (500 ng/mL) and different concentrations of vitamin C (100 µM or 1 mM). EtOH served as control (vehicle). Scale bar, 50 µm. (**E**) Quantification of remaining soluble Aβ40 in the supernatant of platelet culture using Western blot (n = 3).

Platelets are able to modulate soluble, synthetic Aβ40 into forming amyloid aggregates in vitro [15,21,22,23]. To analyze the role of mitochondria in platelet-mediated Aβ aggregation, we treated human platelets with antimycin A (or with EtOH as vehicle) and incubated the cells with soluble, synthetic Aβ40 for three days. Treatment of platelets with synthetic Aβ40 in presence of antimycin A led to increased Aβ aggregate formation in a concentration-dependent manner (Figure 4A,B). The highest aggregate formation was detected in the presence of 500 and 1000 ng/mL antimycin A. Previously it was demonstrated that complex III-derived ROS triggers the formation of Aβ40 by enhanced amyloidogenic amyloid precursor protein processing in HEK293 cells [31]. To investigate whether or not the inhibition of complex III per se leads to the production of Aβ40 in platelets, we incubated platelets with antimycin A for 24 h. However, as shown in Figure 4C, no increase was detected in Aβ40 levels after inhibition of the mitochondrial respiratory complex III (Figure 4C), suggesting that endogenous Aβ40 production does not significantly contribute to Aβ40 aggregate formation in platelet culture.

Aβ40 induces ROS generation in platelets as shown in Figure 1A. To investigate whether or not elevated ROS levels play a role in platelet-mediated Aβ aggregation, we used the antioxidant vitamin C as ROS scavenger. The presence of vitamin C led to decreased amyloid aggregate formation in platelet cell culture (Figure 4D). To confirm this result, we quantified the remaining soluble Aβ40 in the supernatants of platelet cell culture using Western blot analysis. The remaining amount of Aβ40 was increased when platelets were incubated with vitamin C suggesting that ROS generation plays a crucial role in platelet-mediated Aβ aggregation (Figure 4E).

### 2.5. Aβ Induced GPVI-Mediated ROS Production and Integrin α_IIb_β_3_ Activation In Vitro

**Figure 5:**
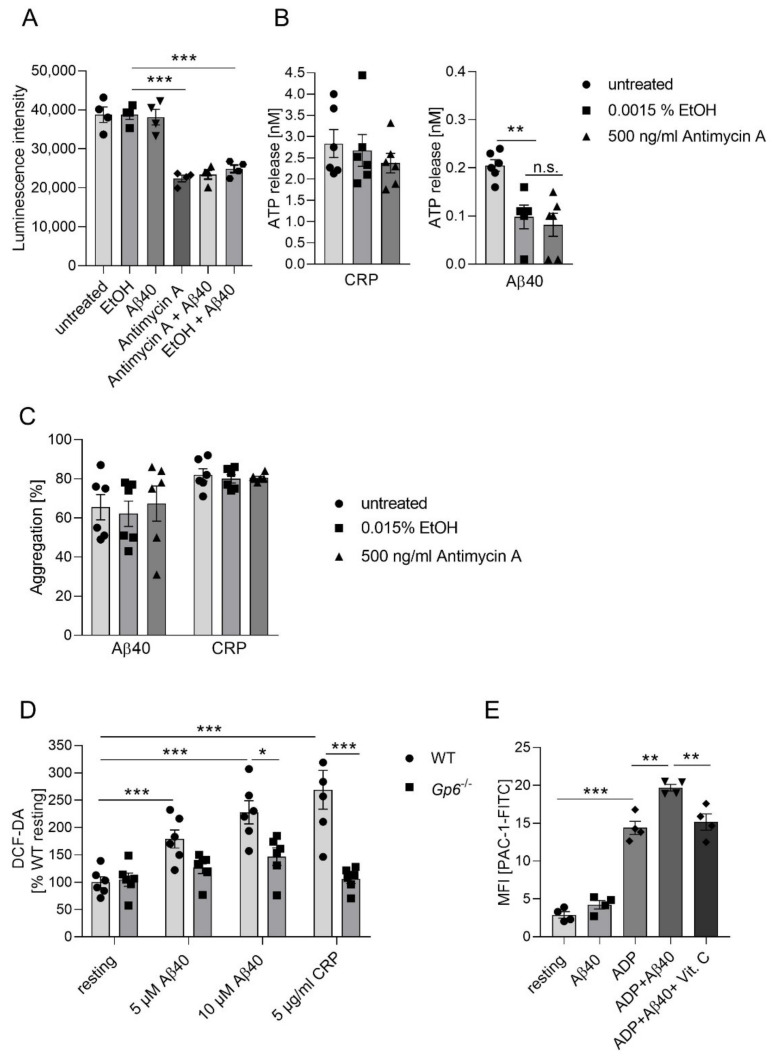
Impact of complex III inhibition on platelet functions following Aβ40 stimulation. (**A**) Detection of intracellular ATP levels after incubation of platelets with Aβ40 (5 µM), antimycin A (12.5 µM) and EtOH (as control, vehicle) for 90 min using Luminescence intensity (n = 4, *** = *p* < 0.001; two-way ANOVA with Tukey’s multiple comparisons test). (**B**) Measurement of ATP release from antimycin A-pretreated platelets (EtOH was used in controls as vehicle) following Aβ40 (20 µM) or CRP (1 µg/mL) (n = 5–6). Analyses were performed using one-way ANOVA and Dunnett’s multiple comparisons post-hoc test. ** *p* < 0.01; n.s.: not significant. (**C**) Aggregation of antimycin A-pretreated platelets upon Aβ40 (10 µM) or CRP (1µg/mL). EtOH was used as control (vehicle) (n = 5–6). (**D**) Measurement of reactive oxygen species (ROS) generation with DCF-DA in GPVI-deficient platelets upon Aβ40 and CRP (n = 6). Data represent mean value ± SEM; two-way ANOVA with Sidak’s multiple comparisons test. *** *p* < 0.001; ** *p* < 0.01; * *p* < 0.05. (**E**) Flow cytometric analysis of integrin activation at the surface of platelets using PAC-1 antibody upon stimulation with Aβ40 (11.5 µM) and ADP (5µM). Where indicated, samples were pre-incubated with vitamin C (1 mM) for 30 min at RT (n = 4). Data represent mean value ± SEM; two-way ANOVA with Tukey’s multiple comparisons test. *** *p* < 0.001; ** *p* < 0.002.

Platelets are metabolically active and display high adenosine triphosphate (ATP) turnover [25]. Luminometric analyses showed a significant reduction of the intracellular ATP level if dysfunction of complex III was induced by antimycin A (Figure 5A). Previously we demonstrated that Aβ40 is able to induce the release of ATP and platelet aggregation [21,23]. Furthermore, ADP/ATP plays an important role in platelet-mediated Aβ aggregation [21] that was enhanced when mitochondrial dysfunction was reinforced by antimycin A (Figure 4A). Therefore, we investigated the effect of complex III dysfunction for Aβ40-induced platelet aggregation and ATP release in the presence of antimycin A. Both, Aβ40 and CRP induced release of ATP from platelets. In the presence of antimycin A, release of ATP was comparable to solvent control EtOH upon CRP (used as positive control) and Aβ40 stimulation (Figure 5B). However, we measured the reduction of the intracellular ATP level (Figure 5A) and of the ATP release (Figure 5B) in response to Aβ40 in the presence of solvent control EtOH. Next, we investigated whether or not the aggregation of platelets is altered by blocking of complex III in platelets. As shown in Figure 5C, platelet aggregation was not altered, either following CRP stimulation or Aβ40 treatment (Figure 5C) when platelets were pre-incubated with antimycin A. Vitamin C was shown to reduce platelet-mediated Aβ aggregation in the presence and absence of antimycin A (Figure 4D), suggesting that ROS generation plays a crucial role in these processes. Moreover, integrin α_IIb_β_3_ and GPVI play an important role in platelet-mediated Aβ aggregation [21,23]. Therefore, we incubated platelets with 5 and 10 µM Aβ40 and determined ROS generation in GPVI-deficient platelets from *Gp6^-/-^* mice. As shown in Figure 5D, ROS generation of GPVI-deficient platelets was significantly reduced in response to Aβ40 and CRP (positive control) (Figure 5D), suggesting that GPVI is involved in ROS-mediated Aβ aggregation facilitated by platelets. ROS production regulates integrin α_IIb_β_3_ activation [32], a major contributor of platelet-mediated Aβ aggregation [21]. Therefore, we next determined integrin activation following Aβ stimulation in the presence and absence of the ROS scavenger vitamin C to investigate, if Aβ-induced ROS production is able to prompt integrin α_IIb_β_3_ activation (Figure 5E). As shown in Figure 5E, pre-treatment with vitamin C led to reduced Aβ40-induced activation of integrin α_IIb_β_3_ in ADP-treated platelets (Figure 5E).

### 2.6. Mitochondrial ROS Production and Mitochondrial Membrane Potential in Platelets from Alzheimer’s Disease Transgenic Mice APP23

**Figure 6:**
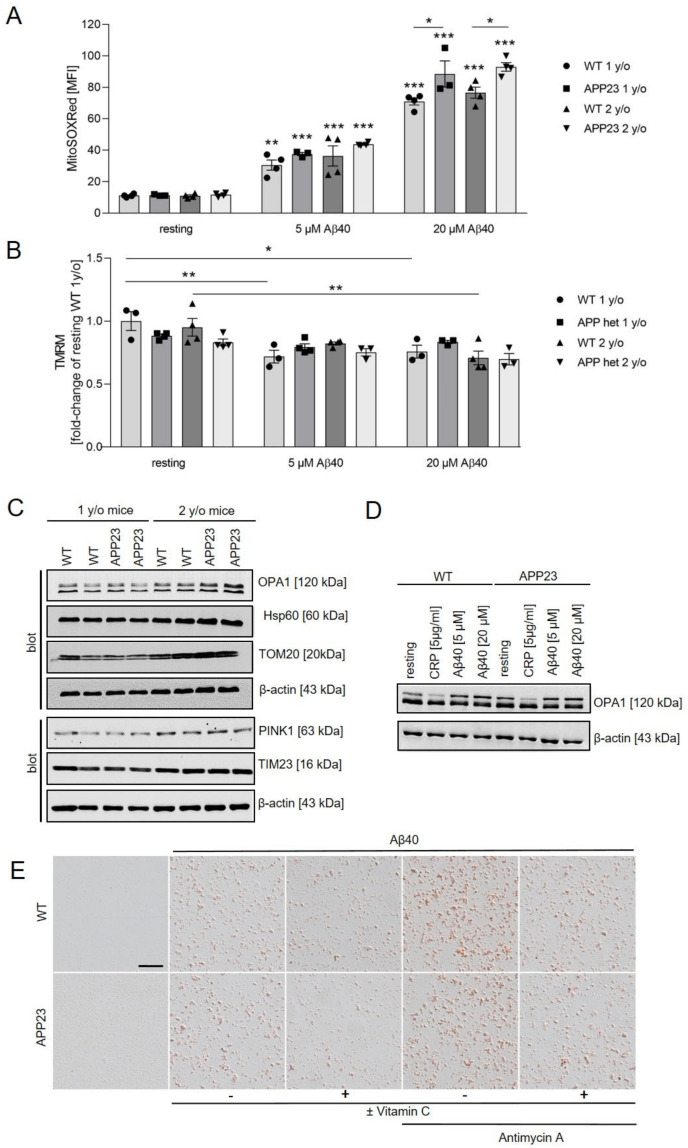
Analysis of mitochondrial function in platelets from APP23 mice. (**A**,**B**) Platelets from one- and two-year-old WT and APP23 mice were incubated with 5 and 20 µM Aβ40 for 30 min. Formation of superoxide was examined using MitoSOX™ Red and depolarization of platelet mitochondrial membrane was observed by decrease in TMRM fluorescence intensity. Data show the mean value ± SEM (n = 3–4), two-way ANOVA with Tukey’s multiple comparisons test. *** *p* < 0.001; ** *p* < 0.01; * *p* < 0.05; vs. basal or as indicated. (**C**) Expression levels of mitochondrial proteins in platelets from one- and two-year-old WT and APP23 mice were determined by Western blot analysis. β-actin served as loading control (n = 4). (**D**) Western blot analysis of the mitochondrial protein OPA1 in platelets from two-year-old WT and APP23 mice after stimulation with Aβ40 and CRP. β-actin served as loading control (n = 3). (**E**) Representative images of congo-red stained amyloid aggregates in the culture of platelets after incubation with soluble, synthetic Aβ40 (5 µM) for three days in the presence or absence of antimycin A (500 ng/mL) and vitamin C (100 µM). Platelets from one-year-old WT and APP23 mice were used (n = 3). Scale bar, 50 µm.

In our previous studies we showed that aged mice (two years old) from the AD transgenic mouse line APP23, which develop amyloid-β deposits in the brain parenchyma and cerebral vessels at this age, exhibit pre-activated platelets in the blood circulation accompanied by enhanced integrin α_IIb_β_3_ activation and degranulation of platelets compared to age-matched control mice [19]. Currently we analyzed mitochondria from APP23 mice using platelets from one- and two-year-old mice. Measurements using the ROS sensitive dye MitoSOX-Red showed that superoxide production in resting platelets from APP23 mice is comparable to resting platelets from WT mice (Figure 6A). The generation of mitochondrial superoxide in platelets was significantly increased when platelets were stimulated with 5 µM Aβ40 and even stronger with 20 µM Aβ40, as compared to resting platelets from one- and two-year-old APP23 and WT control mice. However, stimulation with 20 µM Aβ40 led to increased levels of superoxide in platelets from APP23 mice independent of their age, as compared to age-matched control mice (Figure 6A). The mitochondrial transmembrane potential (Δψm) of non-stimulated (resting) platelets was comparable in platelets from one- and two-year-old APP23 and WT mice (Figure 6B). However, stimulation with 5 µM Aβ40 led to reduced mitochondrial transmembrane potential in platelets from WT mice (Figure 6B). Moreover, stimulation with 20 µM Aß40 reduced mitochondrial transmembrane potential in platelets from one- and two-year-old WT mice (Figure 6B).

Furthermore, we analyzed the levels of mitochondrial proteins in platelets from one- and two-year-old APP23 mice in comparison to age-matched controls. Western blot analysis revealed that expression of TOM20 and TIM23 was increased in APP23 and WT mice at the age of two years without reaching statistical significance. However, no alterations in protein expression was detected between platelets from different groups at the same age (Figure 6C and Figure S3). The eight splice variants and two proteolytic cleavage sites within mitochondrial OPA1 result in long and short forms of OPA1 with divergent functions in cristae biogenesis and mitochondrial fusion [33]. To investigate whether or not Aβ40 induces the cleavage of OPA1 in platelets from WT and APP23 mice, we stimulated platelets with different concentrations of Aβ40 and CRP as positive control. As shown in Figure 6D, protein levels of the long form of OPA1was slightly increased following stimulation of platelets with Aβ40 as compared to resting platelets but does not reach statistical significance (Figure S4). In contrast, the levels of the long form of OPA1 was slightly reduced in CRP-stimulated platelets but did not reach statistical significance (Figure 6D and Figure S4). We next analyzed platelet-mediated Aβ40 aggregation in cell culture using platelets from age-matched APP23 and control mice. As already observed in cultured human platelets (Figure 4), treatment of murine platelets with antimycin A increased the formation of congo-red positive Aβ40 aggregates, whereas treatment with vitamin C reduced the number of Aβ40 aggregates. However, no differences were observed between platelets from APP23 and WT mice (Figure 6E).

## 3. Discussion

This study showed that stimulation of human platelets from healthy donors by Aβ40 led to ROS and superoxide production, reduced mitochondrial transmembrane potential, induced the release of mitochondria from platelets and reduced the content of the mitochondrial protein TOM20. Furthermore, the oxygen consumption rate was reduced when we incubated platelets from healthy donors with Aβ40 or Aβ40 and CRP as deciphered by OCR. Enhanced mitochondrial dysfunction induced by antimycin A led to enhanced platelet-mediated Aβ40 aggregate formation. This was due—at least in part—by GPVI- and ADP-mediated ROS production, leading to enhanced integrin α_IIb_β_3_ activation in the presence of Aβ40. Aβ40 aggregate formation in presence of platelets were comparable between APP23 mice and WT controls, which could be reduced upon treatment with vitamin C.

In line with studies in the past [15], we currently observed that Aβ40 induced ROS and depolarization of the mitochondrial membrane in platelets from healthy donors. In line with previous platelet activation and adhesion studies [22], Aβ1-16 neither induced the formation of ROS and superoxide nor reduced mitochondrial transmembrane potential loss in platelets. This is due to the fact that the RHDS binding sequence of Aβ alone is not sufficient to induce Aβ-mediated alterations in platelets, including Aβ-induced outside-in signaling of integrin α_IIb_β_3_. Thus, Aβ binding to integrin α_IIb_β_3_ and integrin outside-in signaling might be important for superoxide production and dysregulation of mitochondrial membrane potential.

Platelet activation, including granular release and aggregation, are energy-dependent processes. Platelets are able to switch between glycolysis and oxidative phosphorylation using either glucose or fatty acids. Activation of platelets promotes a rapid uptake of exogenous glucose and display a glycolytic phenotype coupled with a minor rise in mitochondrial oxygen consumption [34]. To support platelet activation under nutrient limiting conditions, platelets are able to use glucose, glycogen or fatty acids. Thus, platelets have significant metabolic fuel and pathway flexibility, but mostly use glycolysis for ATP generation upon activation [34,35]. Therefore, we analyzed Aβ40-induced release of ATP and platelet aggregation after inducing mitochondrial dysfunction using antimycin A that inhibits complex III of the mitochondrial respiratory chain. Treatment of platelets with antimycin A results in the reduction of the mitochondrial ATP production and supports ROS generation and mitochondrial dysfunction [36]. Treatment of platelets with high dose of antimycin A results in reduced collagen-induced platelet aggregation and strongly reduced dense granule secretion [37]. Here, we used low doses of antimycin A that is able to amplify platelet-mediated Aβ40 aggregate formation but did not alter platelet aggregation or ATP release, suggesting that ATP content in platelets is still sufficient to allow dense granule release and platelet aggregation.

Platelet-mediated Aβ40 aggregate formation was amplified by mitochondrial dysfunction in a dose-dependent manner using antimycin A (Figure 4 and Figure 6). The reduction of Aβ aggregation by vitamin C strongly indicates that Aβ-induced ROS production in platelets is responsible for platelet-mediated Aβ aggregate formation. Vitamin C is an antioxidant and its protective effects and clinical relevance for AD has been already shown in different studies in the past [38,39,40]. Treatment of human and murine platelets with vitamin C reduced the formation of Aβ aggregates (Figure 4 and Figure 6). In particular, enhanced Aβ aggregate formation following mitochondrial dysfunction by antimycin A treatment was strongly reduced in the presence of vitamin C, demonstrating that the reduction of free radical generation attenuates platelet-mediated Aβ aggregate formation. Thus, enhanced ROS level in AD patients might be critical for platelet-mediated Aβ aggregate formation in cerebral vessels known as cerebral amyloid angiopathy (CAA) [41]. Our data suggests that vitamin C might reduce platelet-mediated effects on CAA even in the presence of already existing Aβ aggregates, because positive effects of vitamin C on the reduction of Aβ aggregates were also observed with platelets from APP23 mice (Figure 6).

Mitochondrial dysfunction resulting in increased ROS generation accounts for platelet-mediated Aβ aggregate formation in vitro. Similarly, in HEK293 cells, mitochondrial ROS production enhanced the formation of Aβ [31]. In contrast to the present study, the authors provide evidence that ROS induced elevated processing of amyloid precursor protein (APP) and that, in turn, Aβ led to mitochondrial dysfunction and increased ROS levels, suggesting a vicious cycle that contributes to the pathology of AD. Here, we observed that ROS is responsible for Aβ aggregate formation but not for the formation of endogenous Aβ by APP processing in platelets. The source of Aβ40 in plasma is highly discussed among researchers. Chen and colleagues believe that platelets are the primary source of amyloid beta-peptide in human blood [14].Wisniewski and Wegiel think that vascular Aβ originates from a different source to Aβ in plaques and is generated locally, principally in smooth muscle cells [42]^.^ The group of M. Jucker believes that, although several factors may contribute to CAA in humans, the neuronal origin of transgenic APP, high levels of Aβ in cerebrospinal fluid and regional localization of CAA in APP23 transgenic mice indicate that neuron-derived Aβ can migrate to and accumulate in the vasculature far from its production site. Thus, Aβ transport and drainage pathways, rather than local production of Aβ by platelets or smooth muscle cells, are a primary mechanism underlying CAA formation [43,44]. Results of our previous studies demonstrate that platelet-mediated Aβ40 fibril formation and aggregation is not altered when we inhibited the Aβ production from APP precursors using inhibitors. This indicates that Aβ40 of platelet origin does not contribute to Aβ40 aggregation in the platelet culture [15].

We have recently shown that GPVI, ADP and integrin α_IIb_β_3_ are involved in platelet-mediated Aβ aggregate formation by direct binding of Aβ to GPVI and integrin α_IIb_β_3_ followed by the release of ATP/ADP [21,23]. Here, our data indicates that GPVI is also responsible for Aβ-induced ROS production and that ROS production is involved in α_IIb_β_3_ integrin activation induced by ADP and Aβ (Figure 5). Integrin activation of platelets by ROS has already been shown earlier [32]. However, the authors used thrombin to produce ROS in platelets but not soluble Aβ as shown here. GPVI-triggered ROS production and enhanced integrin activation might support Aβ binding to integrin α_IIb_β_3_ and to GPVI to reinforce platelet-mediated Aβ aggregate formation. Furthermore, the release of mitochondria following treatment of platelets with soluble Aβ might contribute to enhanced platelet activation and inflammation in AD [30].

Mitochondrial dysfunction has been shown to contribute to the pathogenesis of AD and is responsible for the decrease in respiration as observed in platelets from AD patients [26]. However, Fisar and colleagues found no correlation between dysfunctional mitochondrial respiration and changes in plasma Aβ levels as found in patients with AD [27]. Our data indicates that incubation of platelets from healthy volunteers with Aβ or Aβ and CRP significantly reduced mitochondrial respiration compared to CRP alone, suggesting that Aβ itself is responsible for defects in mitochondrial respiration and that enhanced Aβ plasma levels might affect mitochondrial respiration of circulating platelets in AD transgenic mice and patients.

In AD patients and transgenic mice (APP23), enhanced platelet activation was detected [18,19]. Treatment of APP23 mice with the anti-platelet drug clopidogrel reduced the formation of CAA suggesting that enhanced platelet activation contributes to the pathology of AD [21]. The impact of enhanced platelet activation and mitochondrial dysfunction has already been described in different diseases [45,46,47,48]. Patients with sickle cell disease are characterized by decreased mitochondrial respiration, mitochondrial dysfunction that correlates with enhanced platelet activation and hemolysis both contributing to the pathogenesis of the disease [46]. Dengue infection is accompanied by enhanced activation and mitochondrial dysfunction of platelets [47]. In septic patients, Puskarich and colleagues found a correlation between platelet mitochondrial function and organ failure with increased respiratory rates in non-survivors compared to survivors [48]. In diabetes, hyperglycemia was associated with enhanced collagen-induced platelet activation that was triggered by mitochondrial superoxide production [45]. Furthermore, enhanced levels of ROS induced oxidative stress, which plays a crucial role in tissue damage after brain ischemia/reperfusion [49,50].

Taken together, platelet-mediated Aβ40 aggregate formation is enhanced by mitochondrial dysfunction through GPVI-mediated ROS production and elevated integrin α_IIb_β_3_ activation. Thus, mitochondrial dysfunction contributes to platelet-mediated Aβ aggregate formation, and might be not only a beneficial biomarker but also a promising target to limit platelet activation exaggerated pathological manifestations in AD.

## 4. Materials and Methods

### 4.1. Chemicals, Peptides and Antibodies

Platelets were activated with collagen-related peptide (= CRP, Richard Farndale, University of Cambridge, United Kingdom), synthetic Aβ40 (1-40; Bachem, Switzerland, cat no 4014442.1000) sequence single-letter code (DAEFRHDSGYEVHHQKLVFFAEDVGSN-KGAIIGLMVGGVV), Aβ16 (Aβ1-16, Bachem, Switzerland) ADP (Sigma-Aldrich). Apyrase (grade II, from potato) and prostacyclin from Calbiochem were used for isolation. Antimycin A (Streptomyces sp., A8674-25MG, Sigma-Aldrich, St. Louis, USA) was solved in 95% EtOH. Vitamin C (L(+)-Ascorbic acid) is from VWR Chemicals. Antibodies: Hsp60 (SAB 4501464; Sigma Aldrich, dilution 1:1000), OPA1 (sc-393296, Santa Cruz, dilution 1:500), PINK1 (D8G Rabbit mAb 6946; Cell Signalling, dilution 1:500), TIM23 (BD 611222; BD Biosciences, dilution 1:500), TOM20 (sc-11415; Santa Cruz, dilution 1:500), Amyloid-β (6E10, SIG-39320; Covance, dilution 1:2000). The antibodies β-actin (cat no 4967) and horseradish peroxidase (HRP)-linked secondary antibodies (cat no 7074 and cat no 7076) were from Cell Signaling Technology.

### 4.2. Animals

Heterozygous C57BL/6J-TgN(Thy1.2-hAPP751-KM670/671 NL)23 (APP23) were provided by Novartis Pharma AG. Mice with targeted deletion of GPVI were provided by J. Ware (University of Arkansas for Medical Sciences) and backcrossed to C57BL/6 mice. All animal experiments were conducted according the Declaration of Helsinki and approved by the Ethics Committee of the State Ministry of Agriculture, Nutrition and Forestry State of North Rhine-Westphalia, Germany (reference number: AZ 84-02.05.40.16.073). Mice were maintained in a specific pathogen-free environment and fed standard mouse diet ad libitum.

### 4.3. Murine Platelet Preparation

Platelets were prepared as previously described [23]. Murine blood was taken from the retro-orbital-plexus in a tube containing heparin and centrifuged at 250× g for 5 min at room temperature. Platelet-rich-plasma was obtained by centrifugation at 50× *g* for 6 min and was washed twice with Tyrode’s buffer (136 mM NaCl, 0.4 mM Na_2_HPO_4_, 2.7 mM KCl, 12 mM NaHCO_3_, 0.1% glucose, 0.35% bovine serum albumin (BSA, Sigma-Aldrich, St. Louis, MO, USA), pH 7.4) in the presence of prostacyclin (0.5 µM) and apyrase (0.02 U/mL) at 650× *g* for 5 min at room temperature. Before use, platelet pellets were resuspended in the Tyrode’s buffer (without prostacyclin and apyrase) supplemented with 1 mM CaCl_2_.

### 4.4. Human Platelet Preparation

Platelets were prepared as previously described [23]. ACD-anticoagulated blood was obtained from healthy volunteers between the ages of 18 und 50 years old from the blood bank. Donors provided written informed consent to participate in this study according to the Ethics Committee and the Declaration of Helsinki (study number 2018-140-KFogU). The blood was centrifuged at 200× *g* for 10 min at room temperature. The platelet-rich plasma (PRP) was added to phosphate buffered saline (PBS; pH 6.5, apyrase: 2.5 U/mL and 1 μM PGI_2_) in 1:1 volumetric ratio and centrifuged at 1000× *g* for 6 min. Platelets were resuspended in Tyrode’s-buffer (140 mM NaCl; 2.8 mM KCl; 12 mM NaHCO_3_; 0.5 mM Na_2_HPO_4_; 5.5 mM glucose pH 7.4).

### 4.5. Human and Murine Platelet Culture

Isolated human or murine platelets at the concentration of 2 × 10^6^ platelets/well were added to 150 μL of DMEM medium (Dulbecco’s modified Eagle’s medium, 41965-039; Life Technologies). Platelets were incubated with 5 μM synthetic Aβ40 in the presence of antimycin A (or EtOH as solvent control) and Vitamin C for 3 days at 37 °C and 5% CO_2_. After 3 days, the supernatants of cell culture were collected for determination of remaining Aβ concentration using immunoblot analysis. The supernatants were prepared with reducing sample buffer (Laemmli buffer) and denatured at 95 °C for 5 min. Unbound platelets were removed by rinsing with PBS and adherent platelets were fixed with 2% paraformaldehyde and stained against fibrillary Aβ aggregates with Congo red according to the manufacturer’s protocol (Millipore cat no 101641).

### 4.6. Measurement of Intracellular ROS Level

Washed platelets were added to DMEM medium (Dulbecco’s modified Eagle’s medium) and incubated with different concentrations of Aβ40 or Aβ16 (as control) for 24 h at 37 °C. After incubation, platelets were loaded with 10 µM DCF-DA (2′,7′-Dichlor-dihydrofluorescein-diacetat; D6883; Sigma-Aldrich) at 37 °C for 15 min in the dark. Reaction was stopped using PBS. Samples were analyzed on a FACSCalibur flow cytometer (BD Biosciences).

### 4.7. Measurement of Mitochondrial Superoxide

Washed platelets were pre-incubated with MitoSOX™ Red (M36008; Invitrogen) at room temperature for 30 min in the dark. Then, platelets were stimulated with Aβ40 or Aβ16 (as control) for 30 min at RT. Reaction was stopped using PBS. Samples were analyzed on a FACSCalibur flow cytometer (BD Biosciences).

### 4.8. Measurement of Mitochondrial Membrane Potential

Isolated platelets were treated with Aβ40 for 30 min at RT and then incubated with 100 nM tetramethylrhodamin methyl ester (TMRM; Sigma-Aldrich) in the dark. The reaction was stopped after 30 min incubation using PBS. Samples were analyzed on a FACSCalibur flow cytometer (BD Biosciences).

### 4.9. Measurement of Mitochondria Release Using MitoTrackerTM Green FM

Human isolated platelets were incubated with 100 nM MitoTrackerTM green FM (M7514; Invitrogen)for 30 min at 37 °C under exclusion of light. After incubation, platelets were stimulated with thrombin (0.1 U/mL or 0.5 U/mL) or Aβ40 (2.3 µM or 11.5 µM for 15 min, 1 h and 4 h at RT in the dark. The reaction was stopped with PBS. Samples were analyzed on a FACSCalibur flow cytometer (BD Biosciences).

### 4.10. Cell Lysis and Immunoblotting

Human isolated platelets were stimulated with collagen-related peptide (CRP, 1 µg/mL) or Aβ40 (5 µM or 20 µM) at 37 °C with stirring (250 r.p.m) for 1and 2 h. Stimulation was terminated with 5× ice-cold lysis buffer (100 mM Tris-HCl, 725 mM NaCl, 20 mM EDTA, 5% TritonX-100, complete protease inhibitor (PI) cocktail). Murine platelets were lysed with lysis buffer (15 mM Tris-HCl, 155 mM NaCl, 1 mM EDTA, 0.005% NaN_3_, 5% IGPAL and PI). Cell lysates were prepared by boiling a sample of lysate with sodium dodecyl sulfate (SDS) sample buffer. Platelet lysates were then separated by SDS-polyacrylamide gel electrophoresis, electro-transferred onto nitrocellulose blotting membrane (GE Healthcare Life Sciences). Membrane was blocked using 5% nonfat dry milk in TBST (tris-buffered saline with 0.1% Tween20) and probed with the appropriate primary antibody and secondary antibody HRP-conjugated antibody. Immunoreactive bands were visualized with enhanced chemiluminescence detection reagents using FusionFX Chemiluminescence Imager Systems (Vilber) and quantified using the FUSION FX7 software (Vilber).

### 4.11. Platelet Aggregation

Platelet aggregation was measured as percentage light transmission compared to Tyrode’s buffer (=100%) using Chrono-Log dual channel lumi-aggregometer (model 700) at 37 °C stirring at 1000 rpm. Where indicated, the platelets (20 × 10^3^ cells/µL) were pre-treated with 500 ng/mL antimycin A or 0.0015% EtOH (as solvent control) for 1 h at RT. Platelets were then stimulated with 1 µg/mL CRP or 10 µM Aβ40 and aggregation response was examined.

### 4.12. Measurement of Intracellular ATP Level and ATP Release

Intracellular ATP level was measured using Mitochondrial ToxGlo™ Assay (Promega) following the manufacturer’s protocol. Platelets were adjusted to a final concentration of 2 × 10^2^ and incubated with 5 µM Aβ40 und 12.5 antimycin A (EtOH used as control) for 90 min at 37 °C. ATP release was measured using ChronoLume luciferin assay (ChronoLog) on a LumiAggregometer (model 700, ChronoLog) at 37 °C stirring at 1000 rpm. Where indicated, the platelets (20 × 10^3^ cells/µL) were pre-treated with 500 ng/mL antimycin A or 0.0015% EtOH (as solvent control) for 1 h at RT. After 2 min of incubation with luciferase, platelets were stimulated with 1 µg/mL CRP or 20 µM Aβ40 and monitored for ATP release.

### 4.13. Aβ40 Quantification by Enzyme-Linked Immunosorbent Assay (ELISA)

Platelets were adjusted to a final concentration of 1 × 10^6^/µL and pre-incubated with 500 ng/mL antimycin A or 0.0015% EtOH (as solvent control) for 24 h at 37 °C. Incubation was terminated with 5× ice-cold lysis buffer (100 mM Tris-HCl, 725 mM NaCl, 20 mM EDTA, 5% TritonX-100, complete protease inhibitor (PI) cocktail). After centrifugation, Aβ40 levels in supernatants were measured following the manufacturer’s protocol (human Aβ1-40 ELISA Kit, Cat. No: MBS2506221, MyBioSource).

### 4.14. Measurement of the Oxygen Consumption Rate

Mitochondrial respiration of blood platelets was analyzed by using the Seahorse XFe96 extracellular flux analyzer and the Seahorse XF Cell Mito Stress Test Kit (103015-100, Agilent Technologies), purchased from Agilent (Agilent Technologies, Santa Clara, CA, USA). The Seahorse XF Cell Mito Stress Test Kit allows direct measurement of mitochondrial function in living cells by monitoring the oxygen consumption rate (OCR) and was performed according to the manufacturers’ guidelines. ACD-anticoagulated human whole blood samples were obtained from healthy volunteers. Blood was centrifuged at 200 g for 20 min at 22 °C without brake. The platelet-rich-plasma, PRP thus obtained was separated an added to phosphate buffered saline (PBS, pH 6.5), supplemented with apyrase (2.5 U/mL) and prostaglandin I_2_ (PGI_2_, 1 µM) in a volumetric ratio of 1:10 and centrifuged at 1000 g for 10 min at 22 °C without brake. Washed platelets were subsequently resuspended in Seahorse XF DMEM medium (containing 5.5 mM D-Glucose, 1 mM Na-Pyruvate, 4 mM L-Glutamine, pH 7.4). Afterwards, platelets were seeded in a density of 2 × 10^7^ cells/well into a Cell-Tak (100 µg/mL) coated XFe96 cell culture microplate in Seahorse XF DMEM medium (containing 5.5 mM D-Glucose, 1 mM Na-Pyruvate, 4 mM L-Glutamine, pH 7.4). To evaluate the impact of Aβ40 on mitochondrial respiration using OCR as a readout, the Seahorse XF DMEM medium was either supplemented with 5 µM Aβ40 or without (Bachem, Switzerland). For optimal adhesion, the seeded platelets were centrifuged in two steps at 143 and 213× *g* respectively for one min each. After centrifugation, the cells were incubated for 30 min in a non-CO_2_ incubator at 37 °C, prior the start of the assay. Initially the basal OCR was measured, followed by sequential injections of CRP (5 µg/mL), oligomycin (1 µM), FCCP (0.5 µM) and a mix of antimycin A and rotenone (each 1 µM). ATP-linked respiration is defined as the difference of the last rate measurement before oligomycin injection and the minimum rate measurement after oligomycin injection. Proton leak is defined as the minimum rate measurement after oligomycin minus non-mitochondrial respiration. The OCR measurements were conducted in 3 cycles for each condition. All data were analyzed using the wave software (Agilent Technologies, 2.6.1).

### 4.15. Flow Cytometry Measurement of PAC-1 Binding

Flow cytometry analysis of platelet activation was performed using fluorophore-labeled antibody PAC-1 (activated integrin α_IIb_β_3_ receptor marker, BD Biosciences). A total of 5 µL of whole blood was added to tube containing phosphate-buffered saline (PBS), antibody and agonists (ADP and Aβ40). Where indicated, whole blood was pre-incubated with vitamin C (1 mM) for 30 min at RT. After incubation at room temperature for 15 min, the reaction was stopped by the addition of PBS and samples were analyzed on a FACSCalibur flow cytometer (BD Biosciences).

## Data Availability

The data presented in this study are available on request from the corresponding author.

## Supplementary Materials

The following are available online at https://www.mdpi.com/article/10.3390/ijms22179633/s1.
