# Supplementary material for: Impact of Amyloid-β on Platelet Mitochondrial Function and Platelet–Mediated Amyloid Aggregation in Alzheimer’s Disease
Source: Int J Mol Sci. 2021 Sep 6;22(17):9633. doi: 10.3390/ijms22179633 (PMC8431787; doi:10.3390/ijms22179633)
Supplement: Supplementary file 1 [file ijms-22-09633-s001.zip › ijms-1327780-supplementary.pdf]

Supplemental Figures

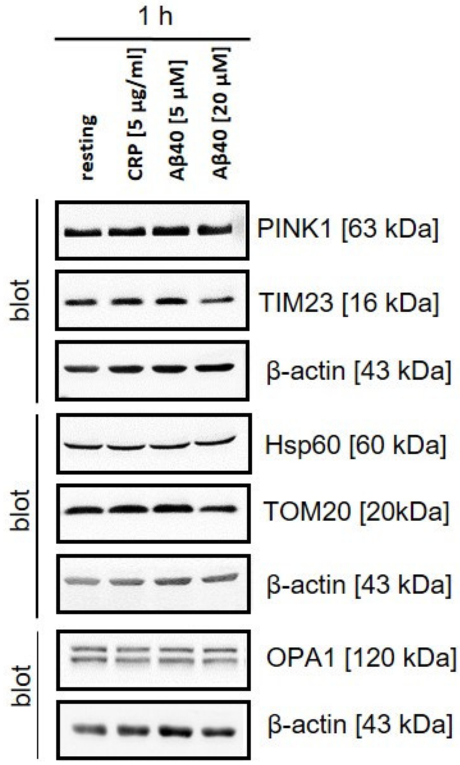

Figure S1: Expression levels of mitochondrial proteins in platelets upon Aβ40 stimulation. Human platelets were stimulated wit 5 µM or 20 µM Aβ40 or 5 µg/ml CRP for 1 h. Using western blot analysis the expression levels of mitochondrial proteins as indicated were detected. β-actin served as loading control (n=4).

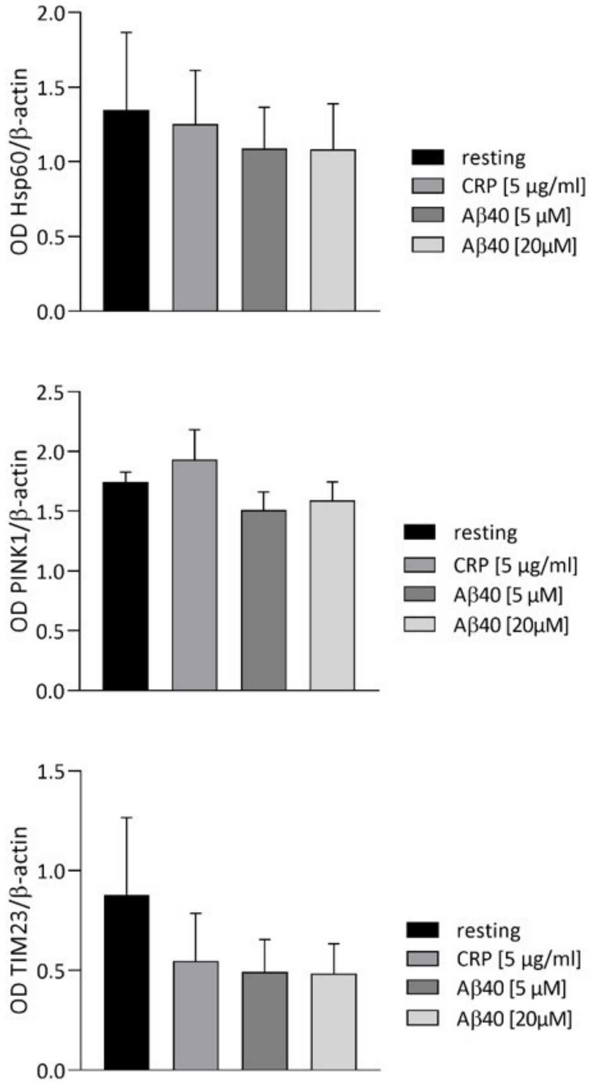

Figure S2: Quantification of expression levels of mitochondrial proteins in platelets upon Aβ40 stimulation. Human platelets were stimulated wit 5 µM or 20 µM Aβ40 or 5 µg/ml CRP for 2 h. Using western blot analysis the expression levels of mitochondrial proteins were detected. β-actin served as loading control. The intensity of bands were analyzed with ImageJ software. Data show the mean value ± SEM (n=4-5).

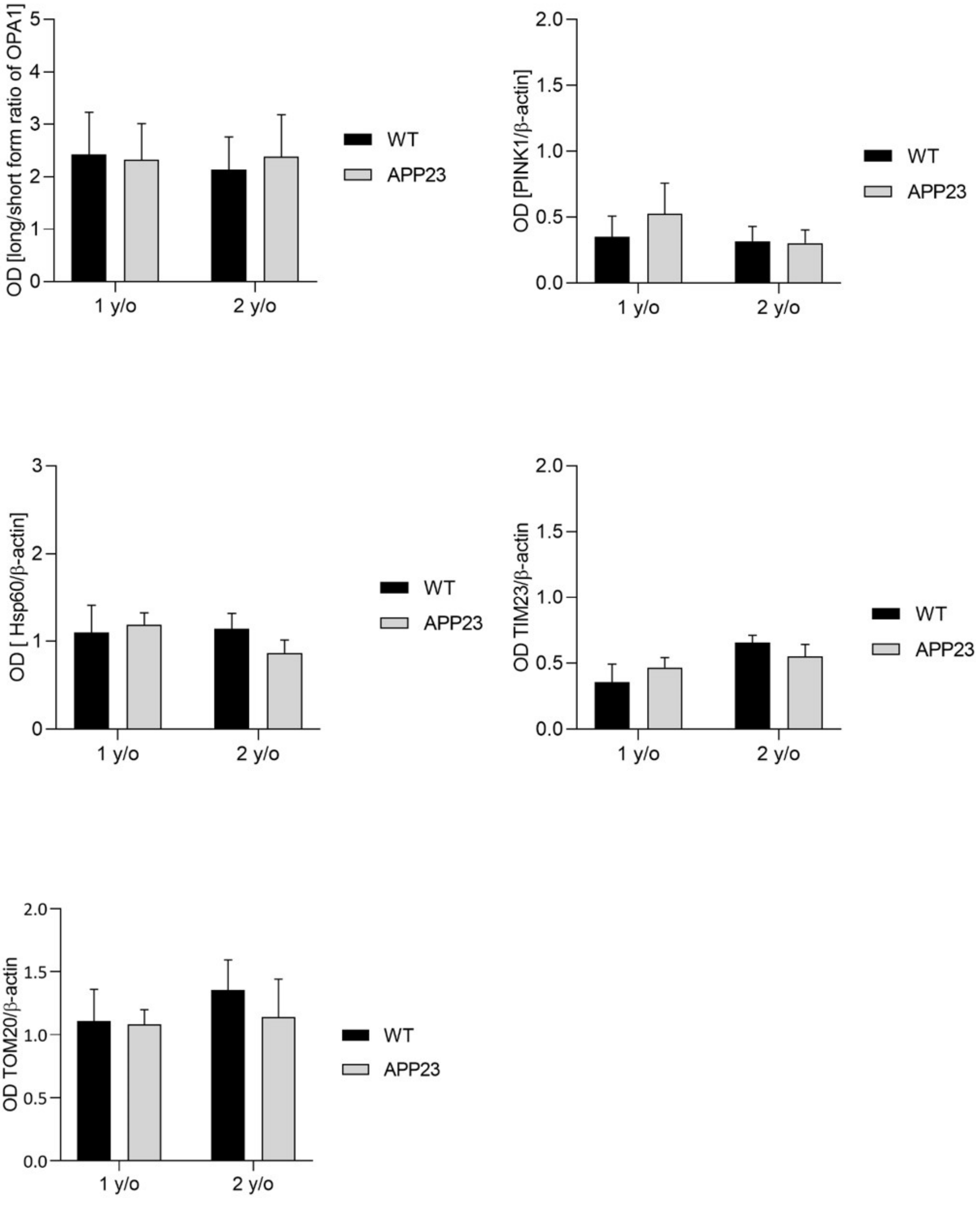

Figure S3: The expression level of mitochondrial proteins in platelets from one year and two year old WT and APP23 mice were quantified by ImageJ software and normalized by the expression of β-actin. Data show the mean value ± SEM (n=4-5).

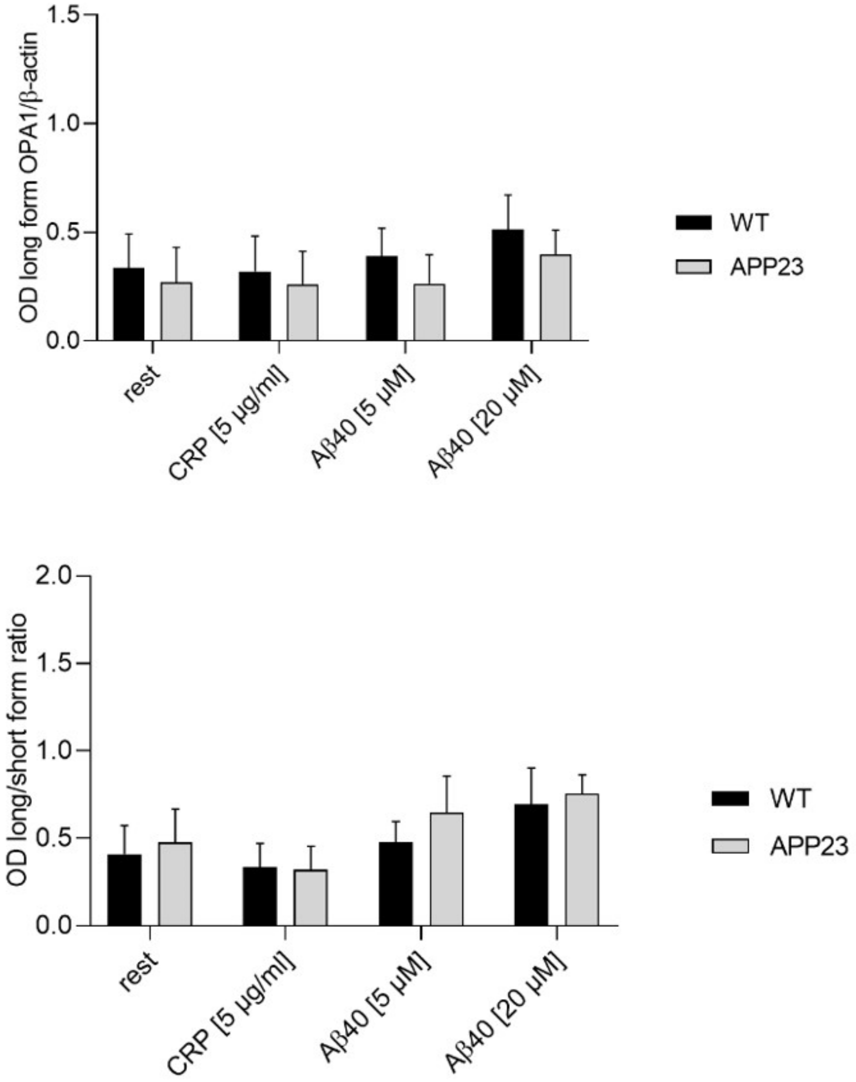

Figure S4: The expression level of mitochondrial protein OPA 1 in platelets from two year old WT and APP23 mice as indicated were determined using Western blot analysis. β-actin served as loading control. The grayscale values of the bands were analyzed with ImageJ software. Data show the mean value ± SEM (n=4-5).
